# Calibrating workers’ trust in intelligent automated systems

**DOI:** 10.1016/j.patter.2024.101045

**Published:** 2024-08-29

**Authors:** Gale M. Lucas, Burcin Becerik-Gerber, Shawn C. Roll

**Affiliations:** 1 USC Institute for Creative Technologies, University of Southern California, Los Angeles, CA, USA; 2 Sonny Astani Department of Civil and Environmental Engineering, Viterbi School of Engineering, University of Southern California, Los Angeles, CA, USA; 3 Chan Division of Occupational Science and Occupational Therapy, University of Southern California, Los Angeles, CA, USA

**Keywords:** trust, automation, calibrated trust, workers

## Abstract

With the exponential rise in the prevalence of automation, trust in such technology has become more critical than ever before. Trust is confidence in a particular entity, especially in regard to the consequences they can have for the trustor, and calibrated trust is the extent to which the judgments of trust are accurate. The focus of this paper is to reevaluate the general understanding of calibrating trust in automation, update this understanding, and apply it to worker’s trust in automation in the workplace. Seminal models of trust in automation were designed for automation that was already common in workforces, where the machine’s “intelligence” (i.e., capacity for decision making, cognition, and/or understanding) was limited. Now, burgeoning automation with more human-like intelligence is intended to be more interactive with workers, serving in roles such as decision aid, assistant, or collaborative coworker. Thus, we revise “calibrated trust in automation” to include more intelligent automated systems.

## The bigger picture

When new systems are deployed, it is critical for employers to have an accurate, theoretically well-motivated model of human trust in AI. Prior work has focused mostly on increasing trust, because users tend to undertrust AI in such contexts. However, such undertrust, that is, trust that is too low, is only half of the problem. Trust can also be too high. Accordingly, we turn to trust calibration, which is an extension of the concept of trust that indicates the degree to which trust is aligned with the actual capability of a technology. Accurate calibration of trust is critical for optimal outcomes in collaboration at work between human coworkers and their automated counterparts, while overtrust or undertrust in technology may be detrimental to team performance in such mixed teams.

## Teaser

Lee and See wrote a seminal piece on calibrated trust, the extent to which the judgments of trust in the automation are accurate. This work adapts their model, modifying it to apply to intelligent automated systems (IASs) operating as coworkers. This paper specifically proposes a new model, CalTruIAS, for calibrated trust-in-IASs to update this understanding of calibrated trust to apply to new technologies with more human-like intelligence and interaction capabilities.

## Main text

Trust in automation has never been a given, even from the very beginning. The first occupational automation was developed in 1785—an automatic flour mill. Until 1840, during the Industrial Revolution, a wave of automated devices that took the role of workers in the workplace was developed. An “automated coworker” is, by our definition, a machine that does a job in the workplace that a human worker had previously done “by hand.” Because of the introduction of such inventions into their occupation, even back then, workers feared losing their jobs and were suspicious of the new devices.

Trust is confidence in a particular entity, the trustee*,* especially regarding the consequences of the entity’s actions. Our definition of trust is broader than other classic definitions^1^ by considering two ways of trusting an entity. The definition includes both (1) trusting that the entity will not exploit a vulnerability (e.g., they will not physically harm me when they very well could) and (2) trusting an entity to complete a task (e.g., I trust” that they can do it, even if the consequences do not involve making me directly vulnerable to the trustee). These are both illustrated further in the section “Calibration of trust.”

In the workplace, trustees can come in various forms, such as other human coworkers, supervisors, the organization, and technological systems. In this paper, we focus on the latter; we generally refer to trust directed at technological system(s) as trust in automation. In this paper, we particularly focus on those technologies that represent intelligent automated systems (IASs) operating as coworkers, doing jobs that were otherwise filled by human workers. As we discuss later, the more general term of trust in automation includes both trust in IASs as well as trust in more mechanical automation. As we describe in depth below, mechanical automation performs work tasks with or without the direct involvement of human workers, but when humans are involved, the technology and human interactions are just that: a machine and a human interacting, rather than like a human interacting with another human. In contrast, IASs infuse human-like intelligence and social capabilities when completing tasks with human coworkers, serving in roles such as assistant or collaborative coworkers. Finally, while we focus on automated coworkers, we do not focus on a particular type of consequence—the consequences such systems can have for the trustor (i.e., worker) include, for example, on performance, as well as physical and mental consequences. Here, we consider all such consequences and do not separate them by these categories.

Until the turn of the 21st century, little theoretical scientific work was developed on the factors that affect trust in automation. To fill this gap, Lee and See^2^ wrote a seminal piece in this area (in fact, the piece is the most cited work on the subject^3^). They adapted established theories of trust between humans to develop a theory unique to trust in automation. While there are several subsequent models of trust in automation, the most prominent are built upon Lee and See’s work and tout it as the core model of trust in automation.^4^^,^^5^ Importantly, Lee and See codified “calibrated trust,” the extent to which the judgments of trust in the automation are accurate.

Lee and See’s model of trust in automation was designed to encompass automation already common in workplaces, such as robotic arms for assembly in manufacturing. In such automation, the machine’s “intelligence” (i.e., capacity for decision making, cognition, and/or understanding) is limited, and neither its appearance nor behavior is intended to be particularly social or human-like. That is to say, this kind of automation was mechanical (compared to the burgeoning automation with more human-like social intelligence). According to Lee and See, automation in “work environments [is] as diverse as aviation, maritime operations, process control, motor vehicle operation, and information retrieval”; this mechanical type of automation that they focused on performed work tasks with or without the direct involvement of human workers, but when humans were involved, the technology and human interactions lacked social aspects.^6^ Mechanical automation was not designed to have human-like social engagements with workers (e.g., through natural language) but rather simply to complete the work tasks. Any exchange of information was on the terms of the automation (direct, task-based exchange), rather than including any forms of social influence, negotiation, rapport building, or the like.

In contrast, the emerging type of more human-like social automaton is intended to interact more with workers, serving in roles such as assistant or collaborative coworkers. Such socially intelligent agents infuse social capabilities such as social influence, negotiation, and rapport building when completing tasks with human coworkers. While new kinds of automation with more human-like intelligence (e.g., programs that run using artificial intelligence [AI]) were being developed at the time, Lee and See did not focus on this type of more intelligent automation. Indeed, according to Chiou and Lee,^7^ their “approach neglects relational aspects of increasingly capable automation.” Scholars have called for deeper consideration of calibrated trust,^8^ and because of its basis in trust between humans, Lee and See’s model is optimized for application to automation that does the job of a coworker. Accordingly, this paper addresses this need by reevaluating the general understanding of calibrating worker’s trust in intelligent automation.

At the outset of this paper, we review Lee and See’s work,^2^ presenting a model of trust in automation based on mechanical automation. Following this, we examine the implications of this model for calibration of trust (i.e., the extent to which the user’s trust accurately reflects the automation’s capabilities). We then expand this model into a new one for calibrated trust-in-IASs (CalTruIAS) in particular by adapting it in a number of ways to reflect subsequent burgeoning developments in technology and society and review our current state of understanding. First, we expand the concept of calibrating workers’ trust to encompass more bases of trust. Second, we adapt Lee and See’s model, modifying to apply to IAS, with an emphasis on the calibration of trust in IAS. We therefore forward this new model, CalTruIAS, for calibrated trust-in-IASs to update this understanding of calibrated trust to apply to new technologies with more human-like intelligence and interaction capabilities. Finally, we consider possible directions for how calibration of trust might evolve in the future, as automation becomes more and more integrated into the workplace. Hence, the paper is organized into three main sections that correspond to these aims: defining calibration of trust in automation, describing our new model for trust-in-IASs, and providing boundary conditions for the work with research implications.

## Defining calibration of trust in automation

### Lee and See’s model of trust in automation

**Figure 1:**
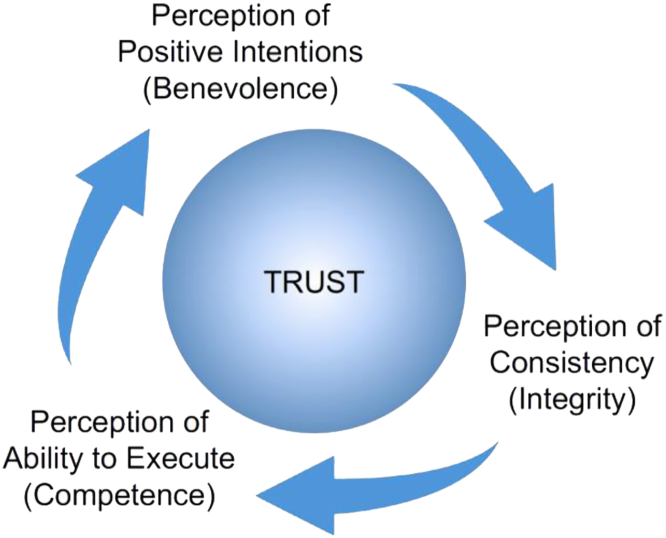
Foundations or bases of trust We trust others based on perceptions of the motivation behind their behavior (benevolence), how well they execute on those positive intentions (ability or competence), and how consistently they execute on those positive intentions (consistency or predictability, which is usually called integrity in these models of trust between humans).

With a focus on the more established mechanical automation of the time, Lee and See^2^ extrapolated the concepts of trust between people to trust in automation. They reviewed the literature on interpersonal trust, establishing that there are three bases for trusting another person (see Figure 1). Lee and See most directly adopted this approach from Mayer, Davis, and Schoorman,^9^ but also demonstrate that other theories of trust between humans map onto the model as well. They say that we trust others based on perceptions of the motivation behind their behavior (benevolence), how well they execute on those positive intentions (ability or competence), and how consistently they execute on those positive intentions (consistency or predictability, which is usually called integrity in these models of trust between humans). According to these scholars of trust between humans, we can trust others (or not) based on their behavior and their intentions to perform the behavior. Starting with such intentions, benevolence-based trust is about the trustee having principles (e.g., “do no harm”) that underlie a trustworthy behavior. Then, competence-based trust is about the trustee having the capability for said behavior. Finally, integrity is about the consistency of that behavior (e.g., personality traits or characteristics that support the trustworthy behavior).

These bases are relevant to trust in (human) coworkers. To illustrate this, we give an example of trusting that a coworker will not harm us when we are vulnerable. One can trust a coworker to engage in safe practices because we know they intend to keep their coworkers safe (e.g., volunteered for the role of safety captain, signaling their motivation to keep coworkers safe; benevolence). However, they may (or may not) be perceived as capable of safe behavior (e.g., unsafe practices due to limited technical capability; competence). Indeed, research has shown that such trust drops quickly in an industrial context when coworkers make operating errors.^10^ Also, their personality may (or may not) be perceived as conducive to always practicing safety behaviors (e.g., unsafe practices due to not being a conscientious person; integrity).

Lee and See^2^ took these three bases of trust and applied labels that are more appropriate for trust in automation: purpose, performance, and process, respectively. They also modified the definitions of the bases accordingly. As with humans, we can trust machines (or not) based on perceptions of the motivation behind their behavior (benevolence/purpose), how well they execute on those positive intentions (competence/performance), and how consistently they execute on those positive intentions (integrity/process).

To illustrate these bases, we give an example of trusting that an automated system will not harm us when we are vulnerable: mechanical automation in the workplace (e.g., mechanical arm on an assembly line, robot moving boxes in a warehouse) would evoke high levels of benevolence/purpose-based trust if workers believe that it was designed to operate without harming them. It would also evoke high levels of competence/performance-based trust if workers think it has the control or dexterity to operate without harming them. In contrast, research has shown that, as with humans, such trust drops quickly in an industrial context when machines make mistakes.^10^ Finally, it would also evoke high levels of integrity/process-based trust if workers believe its programming supports consistently achieving this objective and/or resultant behavior (e.g., has state-of-the-art collision detection with a high degree of accuracy, which quantifies the number of times it is correct out of total tries). That is, integrity/process-based trust comes from believing that the automation can be consistent by having the right “recipe” for the objective or behavior. Then, integrity/process-based trust is based on perceiving the automation as using the right recipe to be able to achieve this objective or behavior, whereas competence/performance-based trust is based on perceiving the automation as capable of successfully executing on that recipe. Therefore, when workers believe that the methods (or recipe) that an automated system uses to operate enable the trustworthy behavior, they experience trust based in integrity/process. Again, all of these are beliefs about trustworthiness, regardless of whether they are based in evidence (indeed, there is a distinction between belief and actual trustworthiness^11^^,^^12^^,^^13^). As we will soon see, trust is calibrated when the beliefs are aligned with actual performance.

Finally, to Lee and See,^2^ because automation lacks intentionality, benevolence does not strictly apply. However, to the extent that the machine is seen as an extension of its human designers (and employers that adopt it, which we call implementers), benevolence becomes more relevant.^14^ A user can make inferences about what the automation would intend (if it could) based on the perceived intentions of its developers. We hold this assumption, and draw conclusions based on it going forward in this paper.

As described above, humans judge others as trustworthy or not based on the belief that they are benevolent in their intentions, competent in their behavior, and exhibit consistency over time (integrity). As implied by the latter basis being judged over time, judgments of trust are not static. They are instead dynamically unfolding over time. We start out by either trusting people immediately (i.e., swift trust) or being suspicious of them, looking for behaviors or evidence that they can be trusted. We prefer to consider a stranger’s behavior, assessing whether they are trustworthy based on their actions (competence; as we do not know if they are benevolent).^1^ However when people focus on the potential benefits that can come from trusting others, they can trust other people without any previous history to base it on.^15^ This swift trust has been demonstrated in laboratory situations designed to key participants into the potential benefits of trusting (i.e., the “trust game,” where if you trust the other participant, you can get more money). Other natural circumstances also lead us to think about the benefits of trust (e.g., asking a stranger for help, letting them introduce you to others).

After some time of observing trustworthy behavior, however, we can develop trust based on integrity. Interestingly, we often continue to trust even when we see behaviors that violate that trust or evidence suggesting that the person should not be trusted (overtrust). This pattern of initial suspicion is the case unless people are forced into a situation where these swift judgments of trust are required (and rewarded) before the trustor is allowed a chance to observe the trustee.^15^ We can have swift trust in others, but we prefer to watch one another’s behavior before deciding if someone is worthy of our trust. As the relationship progresses, integrity of a trustee becomes the core basis of trust, because we can observe how consistent the behavior has been over time, and thus we can make inferences about personality traits or characteristics that underlie this consistency (or lack thereof). Finally, fully mature relationships between humans are based on purpose/benevolence, as we develop faith in teammates, partners, friends, and family over the long term.^2^

While this is generally the trajectory of trust between humans, benevolence-based trust can in fact fluctuate over time as well. Building off the work of Lee and See, other scholars have designated benevolence as one aspect of moral trust; moral trust refers to confidence that a person will choose the morally right thing and not exploit their partner’s vulnerability.^16^ From this perspective, moral trust includes both sincerity, or the belief that the other person is being honest and truthful in their promises, and benevolence, which is their motivation (as Lee and See put it) to not exploit or harm others. Judgments of whether someone is lying (i.e., sincerity) is associated with perception of their facial features, including both emotional and structural cues (i.e., smiles and “baby faces,” respectively, are associated with perceptions that others are being honest^17^^,^^18^^,^^19^^,^^20^). Therefore, in the absence of behavior history that would point to motivation (i.e., benevolence), judgments related to moral trust tend to be more of a gut reaction or determination based on emotional responses (usually to these facial cues). However, that behavior history can accumulate over time. For example, workers would trust their employers gradually as they prove themselves trustworthy through fair treatment (signaling their motivations are good—hence benevolence-based trust). This could occur even if they initially presumed they could not be trusted in this way based on their facial cues,^20^ which would result in benevolence-based trust changing over time.

In contrast, Lee and See^2^ claim that because the attributional process that occurs during judgments of trust is different when the trustee is a machine (compared to a human), the trajectory of trust in automation is different. Attributions of trust in automation are derived from (1) a direct observation of system behavior (competence/performance), (2) an understanding of the underlying methods or recipe (integrity/process), or (3) what the system was designed for (benevolence/purpose). Benevolence/purpose of automation is salient, and even before any interaction, typically seems more transparent to the worker compared to interactions with other humans. Therefore, on the basis of benevolence/purpose, we trust automation more quickly than humans.

In our example of the assembly line or warehouse, workers would tend to assume that the automation was designed to operate without harming them, and thus trust it immediately. In fact, as users assume decent benevolence/purpose, they trust the system to do good even if the developers actually created it for selfish (or even malicious) purposes.^21^ They would assume, that is, that the automation was designed to protect them from harm even if the designers had not considered their safety at all. For example, this may occur when a business adopts a new technology, or even when upgrading the same equipment (one that collects new data that is sensitive, for example). Expectations may be more realistic based on prior use, but we may still assume protection from harm and not realize safety issues such as collecting personal data. Workers should therefore have some skepticism or caution about the developers’ intentions.

When they have not observed the automation’s behavior, nor have other data about the systems’ capabilities, people commonly assume that machines are perfect^4^; we call this the perfect automation schema. Just as with swift trust described above, this may be due to people focusing on the potential benefits that can come from trusting technology (e.g., making a task easier, giving the correct answer when they do not know it). For example, many users of ChatGPT trusted the generated output, even though it was found later to “hallucinate” (e.g., citations) and therefore create faulty content (e.g., false or erroneous sources in references); people may have held such high trust immediately for ChatGPT because they could benefit significantly from using it.

Accordingly, their initial competence/performance trust in automation is often also high.^22^ Because of the assumption of benevolence combined with this initial competence/performance trust, people trust machines highly at the outset. However, as relationships with automated systems progress and actual behavior is observed, and therefore data about the systems’ capabilities becomes available, competence/performance can begin to compete with benevolence/purpose as the basis of trust in automation.^22^ If the automation is highly capable, then trust will remain high; in our example, if the automation seems to have the control or dexterity to operate without harming workers, then people will continue to trust it. If it is not so capable, trust will drop^10^; that is, if workers see it lacking such control, they will change their judgment that it will not harm them.

In summary, we start out by being suspicious of other humans (or trusting them immediately if there is clear benefit to do so), looking for behaviors or evidence that they can be trusted. After some time of observing trustworthy behavior, however, we can develop trust that will even be inviolate to behaviors or evidence suggesting that the person should not be trusted. In contrast, we start our work with machines by assuming that they can be trusted, but any evidence to the contrary may eliminate any chance of trusting a machine to that extent again. Machines can regain trust through better performance (i.e., competence), but once an error occurs, such blind faith in automation is lost.^2^^,^^10^^,^^23^

### Calibration of trust

At any point in the trajectory, a momentary trust judgment can either be properly calibrated or miscalibrated to some extent. Calibration is the accuracy of the trust judgment. This definition by Lee and See^2^ is not only referenced by many other scholars that have come after but it also echoes in subsequent definitions for types of trust. For example, scholars acknowledge that belief in the trustworthiness of automation is distinct from actual trustworthiness (as an objective capability or property of the system^11^^,^^12^^,^^13^); calibrated trust is then the difference between the belief in trustworthiness and the actual trustworthiness of a system.^2^ Using this same distinction, others have defined both warranted^11^^,^^12^^,^^24^ and appropriate^13^ trust in automation as the difference between belief and actual trustworthiness; therefore, warranted trust and appropriate trust are both akin to calibrated trust^24^ (however, for a discussion of the nuanced differences, see Table 1 in Mehrotra et al.^25^).

**Table 1:** The results and affordances of influencing factors

Factor/feature	Task/role	Result	Possible affordance
The mere fact it is automated	decision making, preference ranking, monitor, Q&A/information, route planning, etc.	users trust automation too much, quickly, more than human; belief consistent	can engender trust for high stakes (when system is trustworthy)
Actual reliability	decision making	users more sensitive to moderate or larger differences in reliability	can represent actual consistency in performance
Adjustable automation	decision making, controlling a physical system, etc.	user more likely to trust because this allows for possible collaboration/control	can be used to convey possibilities for error and correction
Human voice with intonation	controlling a physical system	user more likely to trust	can be used to convey more/less certainty
Picture of human expert	decision making (e.g., medical)	user more likely to trust	can cue user in to the developer or operator
More human-like appearance	decision making (e.g., social)	user more likely to trust	can help user feel like it is more/less part of an in-group
More human-like face	decision making (e.g., academic task)	user more likely to trust	can help user feel like it is more/less part of an in-group
Polite personality cues	decision making (e.g., aviation task), monitoring/screening	user more likely to trust	can set norms for further social interaction
Explicit faults or errors	decision making, controlling a physical system, etc.	users trust automation less after fault/errors	can signal “caution” in blindly trusting system
Errors if non-human and seems capable	decision making, preference ranking	contrast effect contributes to greater degree of undertrust	can signal caution in blindly trusting system
Errors if seems human-like	decision making, preference ranking	more forgiving, so less reduction in trust after error	can convey identity as social being, thus deserving more chances
Apology or rationale for error/fault	decision making	more forgiving so less reduction in trust after error	can convey social being deserving more chances
Rapport-building	decision making, preference ranking	sets up for contrast effect if conversation is flawless (otherwise helps to forgive)	can be used to signal system works (yet content could be depicted as fallible)
Acts as if/signals that it is an expert	decision making, route planning, monitoring/screening	user trusts more and relies on recommendations, advice, detection, of system	can engender trust for high stakes (when system is trustworthy)
Explainable AI	decision-making, detection (e.g., medical issue), etc.	user more likely to trust or rely on the system whether or not it is correct	can engender proper trust only when system is trustworthy

Q&A, question and answer.

If the trust judgment is calibrated, then it accurately reflects the system’s capabilities^2^ as well as possibly the purpose and process, which we consider below. Indeed, because Lee and See focused on calibration for competence-based trust, we discuss that first; subsequently, we add to this foundational work by considering calibration for benevolence-based trust and calibration for integrity-based trust, respectively.

### Calibration of competence-based trust

Starting with competence-based trust, when properly calibrated, trust leads users to believe in the system to the right extent and thus use it appropriately. Lee and See also point out that trust can become miscalibrated in one of two directions: overtrust and undertrust (which Lee and See call distrust). Overtrust occurs when the worker trusts the automation more than it merits based on its capabilities, which leads workers to use the system inappropriately (and possibly unsafely). In contrast, undertrust occurs when the worker trusts the automation less than it merits based on its capabilities, which leads workers to underutilize the system.

Again, think about our example of trust in mechanical automation, specifically, manufacturing robots on an assembly line or warehouse (recall that this is an example of trusting that a coworker will not harm us when we are vulnerable). If the worker trusts the robot to be aware of their presence and not collide with them, but, unknown to the worker, this safety feature was not built into the robot and is beyond its capacities, then this would be overtrust; this may lead to injury if the worker is not being cautious enough around the robot. However, if the robot did have this capacity, such trust would be properly calibrated. However, if another worker did not trust the robot’s collision system, then that would represent undertrust, and would lead the worker to keep a wide berth around the robot. Such overly cautious behavior would be unlikely to do any harm in this situation, but one could imagine other cases where it might. While not leading to worker harm, being overly cautious in this instance might still lead to a negative outcome, such as decreasing work productivity.

In addition to considering calibrated trust in mechanical automation, we also offer an example of calibrated trust-in-IASs (and, in contrast to trusting that a coworker will not harm us when we are vulnerable, this example represents trusting automation to complete a task). Imagine a warfighter determining whether they can trust a weapon with automatic targeting. The weapon has IASs that can be queried regarding the shot that is lined up. The warfighter queries the rifle regarding a target at 1,000 yards, and it relays that the shot is possible. If the warfighter trusts the rifle to auto-target at 1,000 yards, but this is actually beyond its capacities, then this would be overtrust; the service member might use the auto-targeting when it should not be used. However, if the weapon could really make such a shot, that level of trust would be accurate. However, if another warfighter—a sniper—did not believe the target detection system worked out that far, but it did, that would represent undertrust and would lead the sniper to try to take that shot unassisted. Such overly cautious behavior could result in the sniper being more likely to miss the shot than if he had adopted IAS.

Additionally, Lee and See^2^ acknowledge that trust in automation can fluctuate. Thus, they define the resolution of trust as the match of the variability in trust judgments to the variability in capabilities. Good resolution occurs when workers have variability in trust that matches the variability of system capabilities; for example, when the robotic coworker on the assembly line or in the warehouse is making errors, they adjust down their level of trust accordingly. However, poor resolution occurs when the variability in trust is less than the variability in capabilities such that in this example, they do not adjust down their level of trust enough when the system is making errors.

### Calibration of benevolence- and integrity-based trust

Lee and See's discussion of calibrated trust implies many of the repercussions that follow trust misjudgments. However, as they focus on the match of trust to an automation’s capabilities, Lee and See’s definition of trust calibration most directly applies to competence/performance-based trust. To broaden the scope of trust calibration, we consider the other bases of trust—benevolence/purpose and integrity/process—as well. Accordingly, trust could also be properly calibrated when it appropriately reflects the system’s intentions and methods/recipe, not just its capabilities.

Considering trust in the manufacturing robot (i.e., trusting it to avoid collision), while benevolence/purpose-based trust results from belief in intention for the robot to avoid collisions, and integrity/process-based trust results from the belief that the intention is reflected in the algorithms that consistently enact this intention, competence/performance-based trust results from the belief (from observation) that the robot executes on the algorithms in a way that results in the intended behavior (i.e., avoids collisions). This flow is illustrated in Figure 1.

First, benevolence/purpose-based trust would be properly calibrated if it appropriately reflected the system’s intentions (i.e., intentions of its developers and implementers). A high level of benevolence/purpose-based trust in the manufacturing robot would be properly calibrated if the designers did intend to keep workers safe in this way. In this case, however, if the worker did not trust the robot, then that would represent benevolence/purpose-based undertrust. In contrast, if programmers did not bother to properly embed such the goal of avoiding collision in the system’s algorithms, then a low level of (or no) trust would be appropriate and thus represent properly calibrated benevolence/purpose-based trust. In this case, however, if the worker instead trusted the robot, then the worker would assume that the intentions were to keep them safe from harm from collisions; this would result in benevolence/purpose-based overtrust. Employees who have such faith in the robot (and/or developers) could end up becoming hurt when its programming (and thus developers) does not live up to those expectations.

Second, integrity/process-based trust is properly calibrated when the level of this kind of trust appropriately reflects the methods that the system uses. Believing the manufacturing robot will be consistent because it has the right recipe to avoid collision represents high integrity/process-based trust. As we explain above, even if the designers did intend for the robot to avoid collisions, the algorithms (e.g., for machine vision, locomotion) may not be made to consistently enact this intention. Employees who believe in the robot this way would have properly calibrated trust if the algorithms were indeed made to consistently avoid collisions, but one who doubts it would have undertrusted the robot. In contrast, the trusting worker would be overtrusting to the extent that the algorithms were not well made to consistently avoid collisions, but the one who doubts it would have more properly calibrated integrity/process-based trust.

Finally, the degree to which trust in automation is calibrated likely systematically differs from trust calibration between humans. There is symmetry to interpersonal trust between humans, in which the trustor and trustee are each aware (to some degree) of the other’s behavior, intents, and trust.^26^ While present, the degree of symmetry may vary across dyads (e.g., less symmetry among dyads disparate in background experiences, worldviews). However, there is no such symmetry in the trust between people and mechanical automation; this leads people to trust and respond to automation differently. This (relative) lack of insight for trustors of automation (compared to trustors of other people) could result in greater mis-calibration. That is, the extent of overtrust or undertrust would, on average, be greater for automation in the workplace than for human coworkers.

### Changes in trust calibration over time

In addition to these differences in calibration of trust based on workers (mis)perceptions of systems’ capabilities, intentions/purpose, and methods/recipe, there are predictable changes in calibration that occur over time. Indeed, as we reviewed above, Lee and See^2^ argue that trust in machines develops differently than trust between people.^27^ These differences in trust development result in different patterns in the extent of (mis)calibration over time. That is, because the development of trust follows different trajectories for trust between humans versus trust in automation, fluctuations in calibration would be different as well. For example, due to the aforementioned salience of purpose, people often exhibit a positivity bias in their trust of novel automated systems,^22^ which can result in initial overtrust based on benevolence/purpose. Recall that on the basis of benevolence/purpose, we sometimes trust automation more quickly than humans (because of the perfect automation schema). Unless workers have some skepticism or caution about the developers’ or implementers’ intentions, this could result in overtrust whereby the workers could be at risk or taken advantage of. In the example of our robotic coworkers, if it were not programmed to prevent worker injury, trusting the robot not to collide with them would represent overtrust, which in this case would be dangerous.

As previously mentioned, competence/performance rises to prominence as a basis of trust in automation as relationships with automated systems progress and actual behavior is observed.^23^ However, because people tend to have a perfect automation schema, where they commonly assume that machines are perfect, their initial competence/performance trust is often also high. If the automation is highly capable, then trust will remain high but would be properly calibrated, as it matches competence/performance of the automation. However, when initial competence/performance trust is too high (not aligned with its actual capabilities), this results in competence/performance-based overtrust as well. If our robotic coworker does not have as much control or dexterity as we think it does, then we may (over)trust it to be more capable of preventing injury than it is. Once this becomes apparent, however, trust will drop, as we mentioned,^10^ and thus again become better calibrated. That is, this trust rapidly dissolves when noticeable errors start to occur, and therefore becomes better calibrated (but sometimes overtrust is replaced by undertrust, as we discuss below). However, this recalibration is limited by the fact that users are more sensitive to moderate or larger differences in reliability and fairly insensitive to smaller ones.^23^

Research has shown that these effects are driven by expectations. Humans have higher expectations of machines’ capabilities than of other humans’ capabilities.^23^^,^^27^^,^^28^ They would think, for example, that the manufacturing robot would weld with greater precision than a human coworker. This also occurs with IASs (as we discuss in the next section). Accordingly, if an automated coworker performs well (e.g., give us correct answers), we would trust the automated coworker more than a human coworker (and thus are more likely to incorporate the information they give us^27^^,^^28^). This could lead to better use of correct answers and subsequent decision making with IASs compared to human coworkers. If the automation is not as capable as we assume, then these higher expectations make workers more prone to competence/performance-based overtrust of IASs than of humans.

Such higher expectations can also make people less forgiving of machines’ errors than humans’. Given that there is a larger contrast effect between expectation (competence/performance-based trust level) and reality (actual capabilities) for automation, this trust is more likely to swing from overtrust to undertrust through calibration, compared to when we revise our judgments of trust in humans. That is, when the robot does not have good control over its movements as not to harm coworkers, which it was expected to have, that causes the initial overtrust to drop precipitously through calibration on down to undertrust; in contrast, because expectations of human coworkers are not as high, there is less of a “backfire” effect when they fail to meet our expectations. We discuss this with respect to IASs in the next section.

Furthermore, users think that automation is objective and consistent (i.e., high integrity/process), while humans are believed to be more changeable (i.e., lower integrity^29^). This might contribute to increased reliance on automation when faced with higher risk decisions^30^; however, if this results in integrity/process-based overtrust, then that decision might not be the correct one. Just as competence/performance-based trust rapidly dissolves when automation errors start to occur, when such a belief does not work out, it could quickly erode integrity/process-based trust, again even to the level of becoming undertrust. If our robotic coworker lacks the state-of-the-art collision detection system that is expected, then integrity/process-based overtrust would drop as well, possibly to the level of undertrust (e.g., believing there is no collision detection system at all when there is one). Another example comes from self-driving vehicles (personal cars and transportation vehicles): surveys of public opinion (e.g., by Pew Research Center) demonstrate that after high profile accidents, people lose trust in autonomous vehicles.

There are opportunities for miscalibrated trust between humans, too, but perhaps less than for trust in automation. Indeed, given that we prefer to watch each other’s behavior before deciding if someone is worthy of our trust, this is less likely to result in the overtrust followed by lowering of trust (to calibration or even undertrust). While overtrust would probably occur less frequently between humans, it could be devastating when it does occur. Indeed, the longer a relationship between humans goes on, overtrust is more likely to occur. Because the basis for trust in other humans shifts to purpose/benevolence over the long term, for such intimate relationships, we are more prone to overtrust in those close to us (compared to machines). Specifically, we have faith in close long-term relationships even if provided with evidence to the contrary; depending on their actual intentions/behavior, this can be appropriately calibrated trust (when they are trustworthy) or overtrust (if they are not). Such overtrust may also persist until the trustor is presented with undeniable evidence to the contrary. This betrayal is particularly painful for the trustor, and can lead to reconsidering, changing, or even terminating the relationship.

Together, these findings on calibration of trust in automation suggest that it fluctuates predictably over time. To summarize, workers start by overtrusting automation because of high expectations, but any other violations of these high expectations lower trust. This could result in proper calibration of trust, or if workers go too far the other way, undertrust. If the latter occurs, then better performance of the machine over time can bring levels of trust back in line with actual capabilities, and may lead to eventual calibrated trust. However, as in human interactions, it is possible to lower trust so far that there is irreversible undertrust in the specific system, developer, or even the type of technology altogether.^31^

## Defining calibration of trust in intelligent automation

The goal of any workplace IASs design should be properly calibrated trust. While designers might be tempted to harness such factors to promote overtrust, this is inadvisable and potentially dangerous. When human workers overtrust automated coworkers, they might not take necessary steps or precautions. This could result in poor work performance, production errors, or even physical harm to workers. Below we cover various factors affecting calibration of trust in IAS; we summarize the impact of these factors or features in Table 1.

### Calibration of trust in IASs

Since Lee and See outlined how people trust mechanical automation, more human-like automation has been developed and become more common.^32^ Recall that interactions between mechanical automation and human coworkers lack human-like social engagement (e.g., through natural language) and instead simply focus on completing the work tasks. In contrast, the emerging type of more human-like social automatons, which we have called IASs, is intended to be more interactive with workers (e.g., serving in roles such as assistant or collaborative coworker). Such socially intelligent agents instead infuse social capabilities such as social influence, negotiation, rapport building, and so forth when completing tasks with human coworkers. A primary example of IASs is conversational AI systems. For illustration, Apple’s Siri includes a number of application services, such as weather and calendar functions, but also has some social responses and jokes (as do other systems such as REA^33^ and SASO^34^).

Compared to mechanical automation, this opens up possibilities for technological systems to have further reach, with positive impacts on users, such as helping workers to be safer and healthier by motivating them to change relevant behaviors.^35^^,^^36^^,^^37^ IASs also have significant implications for the future of work. Akin to the (industrial) revolution described in the Introduction, a new revolution, one fueled by AI, seems imminent.

IASs allow for more complicated relationships with workers, and it is not the same as trust between humans or trust a human has in mechanical automation.^27^ However, trust-in-IASs can still be mapped onto the same three bases as adapted from Lee and See.^2^ In this new model (CalTruIAS), people can likewise trust IASs (or not) based on perceptions of the motivation behind their behavior (benevolence/purpose), how well they execute on those positive intentions (competence/performance), and how consistently they execute on those positive intentions (integrity/process). When people believe that IASs are designed to be beneficial and ethical, it garners benevolence/purpose-based trust. When people believe that IASs’ algorithms or characteristics (i.e., “personality”) support consistently achieving target objectives and resultant desired behaviors, it fosters integrity/process-based trust. Finally, when people believe (or observe) that IASs actually achieve target objectives and exhibit desired behaviors, it breeds competence/performance-based trust. Again, this flow is reflected in Figure 1.

Let us imagine that our robotic coworker on the assembly line or in the warehouse is imbued with AI and has conversational capabilities now. Hence, it fits within the category of IAS. If, in its more human-like interactions with human coworkers (i.e., conversations), the robot signaled that it cared about its coworkers and wants them to not come to harm, then that would boost benevolence/purpose-based trust. If it could also signal that it had the capability to monitor and respond to potential harm (e.g., both by having the control to prevent the accident and verbally expressing that capability, such as a warning), then that would encourage competence/performance-based trust. Finally, if the robot appeared to have a conscientious personality, which would support consistently preventing harm, then it would benefit integrity/process-based trust.

Many IASs are designed to seem trustworthy in these different ways. For example, academic researchers will often brand their IAS with the institution’s logo so that the schools’ positive reputation is evoked. This helps with developing initial trust with workers, as it signals that the system is trustworthy on the basis of benevolence/purpose. Because workers can be more skeptical of the benevolence/purpose of IASs designed by industry (e.g., privacy concerns, selling data), such designers have more to do on this front. The company might use marketing targeted to increase benevolence/purpose-based trust (e.g., using “Privacy. That’s iPhone” to foster trust in Siri). Via marketing (or purposeful design efforts), companies can also go to great lengths to make their IAS seem like (or actually serve as) an expert, as that increases competence/performance-based trust.^23^^,^^38^^,^^39^^,^^40^^,^^41^

Explainable AI, which is designed to make its output interpretable by workers, may result in increased integrity/process-based trust through such transparency about how the IAS makes its decisions.^42^^,^^43^ For example, IASs that make recommendations to workers would be “explainable” if it gave reasons to elucidate those recommendations. Likewise, if a drone with the capabilities of an IAS could explain its course of action (e.g., to investigate movement in sector X), then it would qualify as an explainable IAS. Explainability may be especially helpful when users lack good intuition about how a machine is making decisions.

It is important to note that explainability could increase trust rather than improve trust calibration. Along these lines, Ferrario and Loi^24^ claim that “explainability fosters trust in AI if and only if it fosters justified and warranted paradigmatic trust in AI, i.e., trust in the presence of the justified belief that the AI is trustworthy, which, in turn, causally contributes to rely on the AI in the absence of monitoring.” That is, they restrict the usefulness of explainability to when it fosters properly calibrated (rather than miscalibrated or unwarranted trust). We agree that explainability should only be used to increase trust to the level that is warranted (i.e., calibrated), but it may do so anyway even if it is not warranted, resulting in overtrust due to explainability being seen as signaling a level of trustworthiness beyond the systems’ actual capabilities. Indeed, prior work has demonstrated that explanations increased the chance that humans will trust IASs whether they are correct or not.^44^ So, counter to Ferrario and Loi’s hope, in this case, it was found that explainability increased whether or not it was warranted, thereby increasing judgments of trust, but not trust calibration per se.

However, to the extent that all of these factors do end up promoting increased trust in automation, designers and implementers need to take care not to increase it to the level of overtrust. The goal of system design in terms of trust should always be to strive for properly calibrated trust, for the reasons we explain above.

### Human-likeness of IASs

Many of the findings in this area suggest that the more human-like automation is, the more trustworthy it seems on average. Indeed, research on trust in social agents and/or robots has identified several factors relevant to increasing trust. Much research has demonstrated that the level of automation (LOA) affects trust. For example, users tend to trust IASs with adjustable automation over fixed automation.^45^^,^^46^^,^^47^^,^^48^ That is, fixed automation is static, such as full automation where the IAS has total control and operates without feedback; in contrast, adjustable automation means that the LOA can be changed, for example, from full automation to man-in-the-loop, where the IASs’ actions can be overridden by the worker. This suggests that workers tend to trust systems more if, like humans, the automation demonstrates integrity/process by allowing for some form of collaboration rather than being static (i.e., always in control). Then, both explainability and adjustable automation act as ways to increase trust-in-IASs based on integrity/process. Uniquely, however, adjustable automation in a sense allows the user to adjust the LOA to match their level of trust, giving more control if/when they trust the IAS more.

Increased levels of trust-in-IASs are also associated with a higher level of anthropomorphism. For example, compared to systems that use synthetic speech, IASs are trusted more if they use human speech to communicate with users. However, most of the research on anthropomorphism focuses on the human-likeness of the agents’ appearance. For example, adding a picture of a physician to the interface of a diabetes management application led users to trust it more.^40^ More generally, as IASs are made to appear more human-like, overall trust increases.^49^ Further research has also identified the specific physical features, such as chin shape, that contribute most to this effect.^50^

In addition to physical attributes, the human-likeness of IAS personalities can contribute to trust, especially integrity/process. Research has shown that certain apparent traits can affect workers’ trust. For example, instilling IASs with polite personalities—appearing patient by never interrupting the user—can lead to greater trust.^51^ Researchers have been able to replicate this finding by merely having IASs use more polite speech, thereby increasing trust.^41^ Overall, this research shows that designers can leverage factors (e.g., politeness) that imbue trust between humans to also increase trust-in-IASs.

On average, people trust IASs that are human-like more than systems that entirely lack human features such as conversational ability, voice, appearance, and personality. However, once the IAS appears to be human, turning up the human-likeness from there can actually have a moderating influence on trust. Overall, research has found that once human appearance has been established, increasing the human-likeness of IAS appearance leads to more appropriate calibration, bringing overtrust down. However, counterintuitive findings can result from the combination of other human-like features (e.g., conversational ability, personality) with human-like appearance; we expand our model of trust-in-IASs to consider the combination of these factors below.

When IASs that are human-like in appearance make errors, users do not apply the same inflated expectations that they do with mechanical automation. Giving our (now) intelligent robotic coworker a human-like face would reduce the extent of fluctuations in trust calibration described above; indeed, they would be treated more as a human, with lower expectations, that would help to moderate such fluctuations. Because people are less harsh to other humans, IASs that look more and more human-like are protected more when making errors than mechanical automation, where errors can lead overtrust to flip right over to undertrust; for human-like automation, trust simply becomes more calibrated.^27^

de Visser et al.^52^ followed up on this effect. They found that not only does making IASs appear more and more human breed more calibrated trust by reducing the “hit” that it takes from errors (replicating prior work^27^) but it also does so by lowering initial expectations. Thus, designing IASs with an even more human-looking appearance could also reduce initial overtrust. In summary, making IASs look more human-like—by evoking the lower expectations that we hold of humans—helps to counteract the inflated expectations we have of automation, both at initial trust and later in the face of errors.

The effects of human-like appearance in IASs improve calibration in a number of ways by making the interaction more human-like. While human-like appearance does not initially lead to greater competence/performance-based trust (because it moderates expectations), it can increase trust on average in other ways. Human-like appearance could support the other two bases of trust and thereby help to explain the effect where, on average, IASs with human-like appearance are trusted more. More important, recall that, compared to IASs with other visages, IASs that look human-like take less of a hit to competence/performance-based trust when they make errors, and that overtrust starts out less extreme. Both smaller hits and less initial overtrust reduce the contrast effect that errors cause, thereby preserving a higher average level of competence/performance-based trust. Thus, again, by giving our IAS, the robotic coworker, a human-like face, both processes would contribute to moderating these fluctuations.

Further research with robots has found results that support this model, helping to confirm yet again that once IASs seem human (e.g., act human in terms of good human-like dialog, politeness), human-like appearance can help to reduce these inflated expectations. Indeed, IASs that act very human-like, but don’t look human, can raise expectations even higher. If our robotic coworker had the aforementioned conversational abilities but we did not give it a human-like face, then we would actually have further inflated expectations (instead of reduced), setting us up for larger fluctuations in calibration. Research has considered whether a rapport-building conversation, like you would have with a human coworker about yourself to get to know each other, could help workers trust a mechanical looking IAS (e.g., a robot) in the face of errors.^53^

Recall that when errors start to occur, trust rapidly dissolves and then either gets better calibrated or goes too far over into undertrust. One might expect that having had a bonding conversation (like the one described above) might help workers forgive the mechanical-looking robotic IAS when these errors start to occur. That could, in theory, help prevent undertrust. In fact, Lucas et al.^53^ found that this kind of conversation has the opposite effect. When the robot engaged in the rapport-building conversation before doing a task with workers where it made errors, it was trusted less than when they did not have this conversation first. This resulted in undertrust of the robotic IAS, even though the robot malfunctioned in superficial ways (e.g., repeating answers or requests) and despite its recommendations to the coworker being 100% correct.

Again, the effects of errors such as these (e.g., repeating answers or requests) are usually driven by expectations. Recall that higher expectations make people less forgiving of machines’ errors. The rapport-building conversation went quite smoothly in Lucas et al.,^53^ which raised expectations for workers. Therefore, when workers experienced this effortless conversation that was followed by errors during the task, it effectively dashed their hopes of (and trust in) good performance. Given the strong contrast between initial competence/performance-based trust built through the rapport-building conversation and the subsequent errors, workers’ trust dropped significantly more than among those who did not have the rapport-building conversation first (and thus their expectations were not built up as much).

Additional experimental conditions supported this supposition.^53^ Workers who experienced the rapport-building conversation later, after the task filled with superficial errors, trusted the robot to the same extent as those who did not have this conversation. Because the conversation occurred after the task, it did not set them up for a contrast effect (like those who had the conversation first), and therefore trust was not negatively affected. If anything, having a rapport-building conversation after the task helped to restore any trust that might have been lost due to the errors.

Taken together, the findings of de Visser et al.^52^ and Lucas et al.^53^ confirm that it is the human visage, not social behavior, that triggers us to trust IASs more in the way that we trust humans—have lower expectations and not expect perfection from the start (reducing the perfect automation schema). In fact, good social behavior from a mechanical-looking IAS seemed to increase expectations. Further extrapolating from these sets of findings, once IASs seem human (e.g., act human in terms of good human-like dialog, politeness, and so forth), human-like appearance can help to reduce inflated expectations, but in the absence of human-like appearance, the expectations remain inflated. As alluded to above, we might not want to provide our robotic coworker with such conversational abilities if we keep its fully mechanical appearance (i.e., not make it look more human-like).

Designers can lower initial expectations of IASs, thus reducing competence/performance-based overtrust, by giving them a human-like appearance. They may find this useful for properly calibrating trust, especially when it might make errors (e.g., an untested or beta version). Even if IASs are embodied in robots, they can be made to appear more human-like and will be perceived as more human.^54^ They would therefore have more calibrated trust in the face of errors.^27^ Additionally, it is possible that human social behaviors such as an apology or a rationale for the error could also be helpful at reducing the “hit” that IASs receive after making errors.^22^

In contrast, designers should take care when employing other kinds of social behaviors like rapport-building conversations, especially in the absence of human-like appearance. The level of caution required also depends on the timing of the conversation. While some systems put all the social exchange upfront,^33^^,^^55^ others (e.g., Siri, SASO) interweave the conversational parts into the task itself. There are clear risks, given the above research, to putting the social conversation up front. If the conversation occurs later on, it could even help to restore trust instead of reducing it.

Larger issues arise, however, when we consider the extent to which these kinds of human-like social behaviors result in better calibration of trust. Because the IAS of Lucas et al.^53^ only made superficial errors (e.g., repeating answers or requests), the errors did not reflect a lower capability. Unlike with “real” errors (i.e., that impact performance), competence/performance based-trust should not be lowered by trivial mistakes that are irrelevant to performance; yet, these superficial errors resulted in reductions in such trust-in-IASs, and therefore more poorly calibrated trust, by trusting IASs less than deserved (undertrust).

In contrast, human-like social behaviors like conversations could be used by designers to increase trust to a level of overtrust. Indeed, because rapport-building conversation helped to restore trust when it was used after the task, if IASs are not as trustworthy (i.e., not as trustworthy as in Lucas et al.^53^), such exchanges might increase trust beyond calibrated trust on into overtrust. We thus note again that, while human-likeness may increase trust in IASs, designers and implementers need to take care so not to increase it to the level of overtrust.

## Limitations and future directions in calibration of trust

### Boundaries of new model for trust in IASs

Above, we focused on how human-likeness affects trust mostly in terms of performance. However, it is also possible that making IASs more human-like could result in overtrust based on benevolence/purpose or integrity/process. For example, IASs that appear to have polite personalities might lead to integrity/process overtrust if this politeness does not support the objective (e.g., keeping the human coworker safe). Likewise, through marketing (e.g., “Privacy. That’s iPhone”), designers could try to make IASs seem more trustworthy in terms of purpose/benevolence than they really are, again leading to overtrust.

This latter possibility requires that workers see the machine as an extension of its human designer, so their benevolence/purpose is transferred to the automation. We go beyond Lee and See’s model where, because automation lacks intentionality, benevolence/purpose does not strictly apply. In addition to ascribing benevolence/purpose to IASs based on their designers, it may be the case that IASs are ascribed at least some level of intentionality. Therefore, in their own right, IASs might garner benevolence/purpose-based trust. Indeed, some research suggests that more human-like IASs are perceived to have more intentionality.^56^^,^^57^^,^^58^^,^^59^^,^^60^^,^^61^ Also, recall that because a mechanical trustee is not aware of the user’s behavior, intents, and trust, there is no symmetry when humans trust mechanical automated systems; however, this may not hold for IASs. Accordingly, the IAS might have (or seem to have) more awareness of human interaction partners than mechanical automation. Therefore, workers could perhaps grant benevolence/purpose-based trust to IASs in their own right. To the extent that users believe that IASs have more awareness of the human than they really do, this would likely result in overtrust.

More generally, in this paper, we see some novel ideas generated by reconsidering the work of Lee and See.^2^ However, two major limitations stand out that have not been addressed in this model of trust-in-IASs, which serves as an update to Lee and See’s seminal work. First, the focus is exclusively on automated coworkers that take on the roles of human workers. This ignores the trust in automated tools, such as 3D printers, or sensors, such as those used for heating, ventilation, and air conditioning in the office building, which are not analogous to automated coworkers. Accordingly, for such automated tools scholars might not want to adapt from a model of trust between humans, as such devices likely have different bases for trust than humans do.

Second, little to no attention is paid here to the characteristics of the worker. Various personality variables, demographics, and other individual differences and experiences could lead to differences in how workers respond to automation. The discussion above reflects average workers and ignores how such individual factors would moderate the effects. For example, experience with technology may have a huge moderating effect on the impact of the factors described above. Indeed, experience would influence expectations, which are critical (as we saw) in the (mis)calibration of trust. Future models of trust in automation should include more emphasis on variation on the human side of the equation.

### Future directions

The novel ideas generated by reconsidering the work of Lee and See^2^ suggest possible directions for how calibration of trust might evolve in the future, as IASs become more and more integrated into the workplace. All of these future possibilities involve the context or framing of automation at work.

#### Institutional trust

When unveiling more advanced AI, the context of this emerging technology includes the institution—company, university, design group—that developed it. Accordingly, institutional trust is likely relevant to trust in IAS. We detail this possibility above. Automation may in fact be seen as an extension of its human designers. Thus, workers might assume the intentions of the automation are simply a reflection of the intentions of its creators and/or implementers. Future effort should confirm this possibility and further consider the implications of such a basis of benevolence/purpose-based trust. For example, research could consider how transparency or marketing strategies influence trust (positively and/or negatively).

When the worker really needs the automated system, however, low benevolence/purpose-based trust does not necessarily result in disuse. If a worker does not trust Apple to have their best interests in mind, that may not prevent them from using Siri, especially if they rely on it to meet their needs. Indeed, while many Facebook users were enraged when the company meddled in the 2016 presidential election,^62^ the platform did not see a resultant exodus of users. It met their needs, which drove their use more than the decrement in benevolence/purpose-based trust did. Likewise, IASs that become central to peoples’ lives may be used despite waning institutional trust.

In contrast, the needs of the user may not be as relevant for use in the workplace. The employer may dictate that IASs will be used in the workplace, regardless of what their workers think. This possibility highlights that another kind of institutional trust is also relevant: the extent to which the worker(s) trust the organization that employ(s) them. These institutions enforce (or at least drive) the adoption of IASs in the workplace.

Therefore, workers should also have properly calibrated trust in their organization in regard to the use of IASs. That is, their expectations around the employer’s implementation of IASs should match what the employer is actually doing. This could take several forms. First and foremost, if the employer does not plan to replace workers with IASs, transparency would help reduce undertrust in the automation born out of this fear. Thus, gaps between perceived and real effects of technology on workers’ job opportunities could be a significant contributor to institutional-based undertrust of IASs; future work could consider efforts to quantify the gap and explore its impact on workers.

Second, as employers begin to use technology that automates interpersonal tasks in the workplace (e.g., interviewing, hiring, managing, evaluating, compensating, negotiating), the level of institutional trust will contribute to responses to charging IASs with such tasks. (Of course, procedural fairness, regulation, and ethics of IASs’ processes, thus integrity/process based-trust, will also contribute.^63^)

Third, using IASs to manage human workers could make them feel that they have a lack of control (or autonomy) at work.^64^ Across all three cases, workers may need to have a high level of trust in their employer to prevent undertrust. Future effort should consider the implications of all bases of trust (or not) in both the IASs’ developers and implementers (i.e., employers who are providing the institutional IASs that workers are interacting with when working).

For example, it is possible that if workers trust their employers, they might trust the IAS that they embed in the workplace. If so, employers might be able to harness IASs to improve work, thus potentially further increasing trust in systems. First, workers are more likely to accept behavioral tracking at work when it is run by IASs because it helps workers feel less judged and therefore more autonomous.^65^ Second, IASs can also improve decision making, as teams of AI and humans are better than either teams of only humans or only AI.^66^ Seeing the IAS as a teammate can be critical as well. Unless it is framed just so, people trust automated coworkers (“the AI will serve as your teammate for this task”) more than they do AI assistants (e.g., “the AI is here to assist you with this task”^67^). “Just so” means that only when workers are told that IASs should be given a say in the joint task do they trust AI assistants as much as AI teammates. Accordingly, AI behavioral trackers and AI teammates seem to be trusted, and AI assistants, with their lower level of inherent power,^68^ may require employers to frame their role as more powerful to set the stage for them to be as appropriately trusted. Further endeavors should consider these factors affecting trust when harnessing IASs for behavioral tracking and human-automation partnerships.

While IASs can offer a sense of anonymity,^69^^,^^70^^,^^71^^,^^72^ the trust that workers have that their data will be kept private could be easily violated. That is, while workers might *feel* that the automated coworker will not divulge anything to the employer, for that trust to be properly calibrated, the employer must in fact respect workers’ privacy. Overall, workers report that aside from them being replaced at their job, their greatest concerns around IASs being embedded in the workplace revolve around data privacy and ownership. They want ownership over their data and control over who it is shared with (akin to the Health Insurance Portability and Accountability Act); that way, they can ensure that it will not fall into the wrong hands so as to affect their employment status, insurance coverage, and the like.^73^ As such, if the employer does respect their privacy concerns, then transparency would help reduce undertrust in the automation stemming from such concerns. However, if the employer does not take these needs as seriously, properly calibrated (i.e., lower) trust would result from knowing that the data IASs collect could be used against them or sold by the employer for a profit. Future effort should address workers’ concerns regarding employment security and data privacy in a transparent manner to reduce undertrust in IASs in the workplace. More generally, future studies should also consider how public policies that regulate automation (e.g., privacy regulation) impact trust-in-IASs.

Finally, introducing IASs into the workplace may have significant impacts on the human relationships that already exist. Adding a human coworker to an established team can change the group dynamics; even more so, there are likely to be ripple effects of introducing automated coworkers. This raises many questions about the possible social repercussions of introducing IASs at a worksite. As poignantly posed by Glikson and Woolley,^32^ “for example, how does the implementation of AI-guided hiring and evaluation change the relationships of workers with their jobs? With their coworkers? With their supervisors? How does it shape the distribution of power in the organization?” These effects need to be studied to understand the fuller ramifications of adding IASs to the workplace.

#### Framing IASs in the workplace

Other possible directions to consider are more about the framing of IASs by employers and/or developers for workers. Primarily, to the extent that the employer wants to foster the proper use of IASs, it needs to be framed in ways that encourage workers to better calibrate their trust judgments. This would require employers to help workers to have accurate perceptions of IASs’ intentions (i.e., the developers or implementers), capabilities, and methods/recipe. Education about IASs could therefore become an important means to help workers to properly calibrate their trust judgments. This may be particularly necessary when the system is not explainable, as discussed above.

Additionally, the visibility of IASs may have a similar impact. While explainability is about the transparency of the reasons (e.g., for IASs’ judgments, recommendations, and so forth), visibility is about awareness that IASs are operating. Indeed, IASs may be part of a larger, complex system and users may not be cognizant of the fact that the particular IAS is running in the background. Future research should consider how trust in these more complex systems is developed.

Future work also needs to consider how the experience of working with IASs impacts our general view or framework about automation, and thus may change our trust in both automated and human coworkers. Technology that is well established (e.g., manufacturing robot in automotive assembly line) is likely to be perceived as reliable—and rightfully so—thus resulting in better calibrated trust. Indeed, new IASs tend to be less consistent, given the newer, evolving technology; therefore, while it might make sense for workers to trust a manufacturing robot to be more precise than a human coworker (exhibit properly calibrated trust), high levels of trust in IASs (based in the still-high expectations) may represent overtrust in these nascent and thus more error-prone technologies.

Also along these lines, as workers consistently build experience with IASs in the workplace, some of the overall trends described above may change. For example, with more experience with IASs, we may not have such high initial (over)trust of it; viewing such automation as “perfect” may no longer be the default for workers, suggesting that the perfect automation schema may be reduced in such cases. It may also, for instance, raise our expectations of human coworkers in certain areas, such as areas where workers observe that humans outperform machines. However, if IASs can really evolve to become truly human-like, then perhaps that will obviate our model of trust-in-IASs. In a case that we treated humans and human-like AI the same, we might be able to apply models of trust between humans. It is also possible that, one day, we might trust IASs more than humans.

Finally, IASs can be framed as machines, or a system can instead present itself as if it is a human. The latter framing is often implemented through backstories that portray them as human (“I was born in the United States”) rather than automated (e.g., “I was created at an AI development company”). IASs are perceived equally trustworthy if they use a human backstory while having a rapport-building conversation as if they speak about themselves being a machine.^74^ Thus, a system’s portraying itself as human may not hurt trust-in-IAS, and, if his portrayal evokes a sense that it is human in the same way that human-like appearance does, then it could help with calibrating trust (as described above).

While this work suggests that workers accept IASs speaking as if they were a real human, there are likely to be some limitations to this. For example, in the context of physical tasks, workers might not appreciate encouragement that includes statements such as “I did it” from IASs pretending to be human, as disembodied IASs lack the ability to do anything physical at all.^75^

Moreover, scholars and developers in this area should consider other personas that IASs can adopt, from animals^76^ to buildings,^77^ and how such personas can improve the calibration of trust (and thus subsequent use of IASs). Just as human-like appearance can help set better expectations, perhaps other kinds of personas could help establish trust that is even more finely calibrated.

For example, when the personal computer (PC) was created, developers made the interface look like a desk to rein in expectations of its capabilities; that way, it would be seen as a tool, not an intelligence. Likewise, we find that IASs benefit from human-like appearances because they help to counteract the otherwise inflated initial expectations workers have of automation. However, it is unclear how to similarly improve the calibration of trust as IASs become more advanced, and thus (perhaps) more trustworthy. Who or what should IASs resemble to support high levels of trust, which would be properly calibrated trust given the strong capabilities of such advanced systems?

Indeed, while we have come a long way from bringing PCs into the home and workplace, and even longer from the first automation in the workplace, as more and more advanced IASs are developed, society is facing this level of challenge again. Fortunately, we can draw upon and update theories that served us in prior stages, and use our past adaptations to inspire future innovations to meet our emerging needs.
